# Association of maternal diet with human milk fatty acid and macronutrient composition: a Saudi cohort study

**DOI:** 10.3389/fnut.2026.1830854

**Published:** 2026-05-04

**Authors:** Shahad Alodhaybi, Manal Abdulaziz Binobaed, Rasha Homoud AlAnazi, Nora Elwehedy, Muneera Baraja, Fatimah Yousef Aljawoan, Waleed Tamimi, Lamia Mohammed Elamin, Azza Madkhali

**Affiliations:** 1 Department of Food Science and Nutrition, King Saud University, Riyadh, Saudi Arabia; 2 Security Forces Hospital Program, General Director of Medical Services, Ministry of Interior, Riyadh, Saudi Arabia; 3 Pediatric Clinic, Ministry of National Guard Health Affairs, Riyadh, Saudi Arabia; 4 King Abdulaziz Medical City, King Abdullah International Medical Research Center, King Saud bin Abdulaziz University for Health Sciences, Riyadh, Saudi Arabia; 5 Family Medicine, Ministry of National Guard Health Affairs, Riyadh, Saudi Arabia; 6 Dent Medical Center, Riyadh, Saudi Arabia; 7 Breastfeeding Association, Riyadh, Saudi Arabia; 8 Laboratory Medicine, Ministry of National Guard Health Affairs, Riyadh, Saudi Arabia; 9 Obstetrics and Gynecology, Ministry of National Guard Health Affairs, Riyadh, Saudi Arabia

**Keywords:** fatty acid composition, human milk, lactation, maternal diet, omega-3, Saudi Arabia

## Abstract

**Introduction:**

Maternal nutrition influences human milk (HM) composition, particularly its fatty acid (FA) profile. However, evidence from Middle Eastern populations, including Saudi Arabia, remains limited. This study aimed to investigate the association between maternal diet and HM composition in a Saudi population.

**Methods:**

In this secondary analysis of a prospective cohort of Saudi breastfeeding mothers, a subsample of 40 women was selected. Maternal characteristics, dietary intake, and HM samples were collected between 4 and 13 weeks postpartum. Dietary intake was assessed using a 24-h dietary recall and a food frequency questionnaire. HM macronutrients and FA composition were analyzed, and associations with maternal dietary intake were examined using correlation analyses.

**Results:**

HM macronutrient concentrations were relatively stable and showed minimal associations with maternal dietary intake, except for a positive correlation between maternal monounsaturated FA intake and milk protein (*r* = 0.384, *p* < 0.05). In contrast, HM FA composition—particularly omega-3 (n-3) polyunsaturated FAs—was significantly associated with maternal diet. Maternal energy intake was positively correlated with milk eicosapentaenoic acid (*r* = 0.527, *p* < 0.01) and total n-3 (*r* = 0.489, *p* < 0.01) and inversely correlated with the n-6/n-3 ratio (*r* = −0.410, *p* < 0.01). Maternal n-3 intake was positively correlated with corresponding HM n-3 levels.

**Conclusion:**

Distinct dietary patterns observed in this Saudi cohort, potentially shaped by cultural traditions during pregnancy and the postpartum period, may contribute to variability in HM FA composition. In this sample, both maternal n-3 intake and HM n-3 levels were low compared with global averages, suggesting a potential role for culturally appropriate dietary strategies to improve maternal and infant n-3 status.

## 1 Introduction

Human milk (HM) is a nutritious fluid with complex composition that primarily provides water, lactose, fat, and easily absorbed proteins, alongside vitamins and minerals, to meet infant energy and nutrient needs. Typically, it includes 87% water, 6%–8% lactose, 3%–5% fat, and 1%–2% protein (1–3). HM also contains numerous bioactive, non-nutritive components—such as hormones, growth factors, immune cells, immunoglobulins, oligosaccharides, microRNAs, and resident microbes—that play key roles in immunity, growth, and microbiota development rather than serving as direct energy sources (4, 5).

HM composition is highly variable and is influenced by a broad range of maternal, infant, physiological, and methodological factors (6). Among maternal determinants, diet is considered particularly important and has been extensively studied because it can be modified through targeted interventions. Current diet, long-term dietary habits, and supplement use contribute to variations in HM composition, especially in the fatty acid (FA) profile. Intervention and observational studies show that higher maternal fat intake or high-fat dietary patterns are associated with increased total fat and altered FA composition in HM, whereas diets richer in vitamins, minerals, and fiber, and higher intakes of omega-3 (n-3)-rich foods are linked to higher levels of n-3 FAs in the milk (7–10). Not only current intake but also habitual diet before and during pregnancy has been associated with HM FA composition (9, 11, 12). In addition, n-3 supplementation during lactation can rapidly and substantially modify the HM FA profile (13, 14). Most of this evidence is derived from European, North American, and Asian populations (9, 15, 16). Data from Middle Eastern populations are lacking despite their distinct dietary patterns.

Therefore, this study aimed to investigate the association between maternal diet and HM composition in a Saudi population.

## 2 Materials and methods

### 2.1 Study design and participants

This study is a secondary analysis of a prospective cohort of Saudi breastfeeding mothers. The original cohort study investigated the impact of maternal hypothyroidism on HM macronutrient and FA composition (17).

For the present analysis, a sub-sample of 40 mothers was selected to evaluate the association between maternal nutrition and milk composition. From the original cohort, mothers with uncontrolled hypothyroidism were excluded from this secondary analysis. In addition, one mother who had a history of hemi-thyroidectomy but normal thyroid function tests, and was therefore not included in the original analysis, was added to the present sub-sample.

In total, 30 euthyroid controls, 9 mothers with well-controlled hypothyroidism, and 1 mother with a history of hemi-thyroidectomy and normal thyroid function were included (*n* = 40). Recruitment procedures and full inclusion and exclusion criteria for the cohort have been described previously (17).

### 2.2 Maternal characteristics and dietary information

Maternal characteristics including (demographics, medical history, and infant feeding practices) were collected via a structured questionnaire. In addition to dietary intake. Maternal height and body composition (weight, fat percentage, muscle mass, body mass index [BMI]) were assessed using standardized stadiometry and bioimpedance, with pre-pregnancy BMI derived from recalled, record-verified weight and classified per CDC criteria; infant weight, length, and head circumference were measured by a pediatric nurse using calibrated digital scales, a length board, and a non-stretchable tape.

Dietary intake was assessed using two methods: a 3-day 24-h dietary recall and a modified food frequency questionnaire (FFQ). The 24-h recalls (two weekdays and one weekend day) were used to estimate energy, macronutrients, total FA intake. Data were analyzed using ESHA Food Processor software (ESHA Research, Salem, OR, USA), which is based primarily on the United States Department of Agriculture (USDA) National Nutrient Database. Traditional Saudi dishes were broken down into ingredients and matched to the closest available items in the ESHA database, informed where possible by Saudi food composition data.

The FFQ was adapted from Herter-Aeberli et al. (18), a previously validated questionnaire for assessing n-3 intake. The original FFQ was expanded to better reflect local dietary habits and to include additional foods rich in total fat and omega-6 (n-6) and n-3 polyunsaturated FAs (PUFAs).

The modified FFQ was used in two manners:

1. Quantitative omega-3 intake:

Using only the food items from the original validated FFQ of Herter-Aeberli et al. (18), we estimated daily n-3 intake. FA content in these foods was obtained from the USDA National Nutrient Database, and daily intake was calculated from reported frequency and portion size. This estimated n-3 intake was then correlated with HM n-3 concentrations.

2. Frequency of fat-rich foods:

Using the full modified FFQ (original items plus added fat-rich foods), we assessed the frequency of consumption of selected fat- and n-3–related foods and examined their associations with HM macronutrient and FA composition.

### 2.3 Sample collection and analysis

HM samples were collected between 4 and 13 weeks postpartum, between 9:00 a.m. and 3:00 p.m., at least 2 h after the last nursing event, using a hospital-grade electric breast pump (Symphony®, Medela) to reduce variability. Samples were immediately aliquoted and stored at −70 °C until analysis.

Fat extraction and esterification were performed using the gravimetric method described in ISO 1736:2008(E) / IDF 9:2008(E), with minor adaptations for liquid milk (19). FAs were then quantified using gas chromatography with flame ionization detection. HM macronutrients (fat, protein, carbohydrate) and total solids were measured using a mid-infrared milk analyzer (Miris Human Milk Analyzer; Miris AB, Uppsala, Sweden). The complete analytical protocol has been described in detail elsewhere (17).

### 2.4 Statistical analysis

Statistical analyses were performed using IBM SPSS Statistics software (version 27.0; IBM Corp., Armonk, NY, USA). Data are presented as mean ± standard deviation (SD) unless otherwise indicated. Pearson’s correlation coefficient was used to assess associations between maternal continuous dietary intake variables and HM components. For the fat-rich FFQ, Kendall’s τ_b correlation was applied to examine associations between ordinal frequency of intake variables and milk composition. For group comparisons involving 2–4 groups, non-parametric tests (Mann–Whitney U test for two groups and Kruskal–Wallis test for more than two groups) were used due to the small group sizes and non-normal data distributions. A *p*-value <0.05 was considered significant.

## 3 Results

### 3.1 Demographic characteristics

This study cohort included 40 Saudi women and their term infants. Most women were older than 25 years, had university education (52.5%), were unemployed (87.5%), and were overweight or obese (72.5%). More than half of the mothers reported not exclusively breastfeeding (60%) and having history of breastfeeding (77.5%). Of the 40 women 9 had an endocrine disease (controlled hypothyroidism) and 6 gestational diabetes (GD) (Table 1).

**Table 1:** Maternal and infant characteristics of the study sample (*n* = 40).

Characteristics	Saudi mothers (*n* = 40)
Maternal age (years)	
20–25	4 (10.0%)
26–30	7 (17.5%)
31–35	13 (32.5%)
36–40	11 (27.5%)
>40	5 (12.5%)
Education	
Not educated	2 (5.0%)
Highschool or less	14 (35.0%)
Diploma	2 (5.0%)
University education	21 (52.5%)
Higher education	1 (2.5%)
Employment status	
Employed	5 (12.5%)
Not employed	35 (87.5%)
Household income (SR)	
<5,000	2 (5.0%)
5,001–10,000	19 (47.5%)
10,001–15,000	13 (32.5%)
>15,001	6 (15.0%)
Pre-pregnancy BMI	
Healthy (18.5–24.9 kg/m^2^)	11 (27.5%)
Overweight (25–29.9 kg/m^2^)	19 (47.5%)
Obese >30.0 kg/m^2^	10 (25.0%)
Parity	
1 child	7 (17.5%)
2 children	8 (20.0%)
3 children	6 (15%)
4 + children	19 (47.5%)
GDM	
Yes	6 (15.0%)
No	34 (85.0%)
Endocrine diseases	
Yes	9 (22.5%)
No	31 (77.5%)
Mode of delivery	
Vaginal	30 (75.0%)
Caesarean section	10 (25.0%)
Feeding pattern	
Exclusive	16 (40%)
Mixed	24 (60%)
History of breastfeeding	
Never	9 (22.5%)
<6 months	9 (22.5%)
6–11 months	4 (10.0%)
12–18 months	7 (17.5%)
2 years or less	11 (27.5%)
Infant sex	
Boy	16 (40.0%)
Girl	24 (60.0%)
Infant herb intake	
Yes	12 (30.0%)
No	28 (70.0%)
Knowledge of omega-3	
Yes	34 (85.0%)
No	6 (15.0%)
Omega-3 during pregnancy	
Yes	6 (15.0%)
No	34 (85.0%)
Omega-3 supplements (last 4 weeks)	
Yes	2 (5.0%)
No	38 (95.0%)
Multivitamin supplement	
Yes	21 (52.5%)
No	19 (47.5%)

Data are *n* (%). GD, gestational diabetes; BMI, body mass index.

### 3.2 Human milk macronutrient and fatty acids

Table 2 presents mean milk energy and macronutrient content in Saudi women. The mean energy content was 72.88 ± 15.39 kcal/100 mL. The mean concentrations of carbohydrates, fat, and true protein concentration were 7.70 ± 0.51 g/100 mL, 3.93 ± 1.64 g/100 mL, and 1.07 ± 0.41 g/100 mL, respectively. The FA composition is presented in Table 3. The proportions of FAs in HM were 45.75 ± 6.16% saturated FAs (SFAs), 36.86 ± 4.82% monounsaturated FAs (MUFAs), 16.48 ± 3.09% PUFAs, and 0.31 ± 0.21% trans FAs (TFAs). Oleic acid was the most abundant FA in HM 32.30 ± 4.03%, followed by palmitic acid 25.50 ± 2.49% and linoleic acid (LA) 14.30 ± 2.94%. Total n-3 FAs were 0.51 ± 0.19%, and docosahexaenoic acid (DHA) was 0.08 ± 0.04%.

**Table 2:** Mature human milk macronutrient composition and energy content in Saudi women (*n* = 40).

Human milk macronutrients and energy	Mean ± SD
Carbohydrates (g/100 ml)	7.70 ± 0.51
Fat (g/100 ml)	3.93 ± 1.64
True protein (g/100 mL)	1.07 ± 0.41
Crude protein (g/100 mL)	1.33 ± 0.52
Total solids (g/100 mL)	13.11 ± 1.72
Energy (kcal/100 mL)	72.88 ± 15.39

Data are presented as Mean ± SD.

**Table 3:** Mature human milk fatty acid composition (% total fatty acids) in Saudi women (*n* = 40).

Fatty acids %	Mean ± SD	Fatty acids %	Mean ± SD
Total SFAs	45.75 ± 6.16	C18:2 n-6 (Linoleic acid)	14.30 ± 2.94
C6:0 (Hexanoate)	0.02 ± 0.02	C18:3 n-6 (*γ*-Linolenic acid, GLA)	0.13 ± 0.05
C8:0 (Octanoate)	0.13 ± 0.06	C18:3 n-3 (α-Linolenic acid, ALA)	0.37 ± 0.15
C10:0 (Decanoate)	1.33 ± 0.41	C18:4 n-3 (Stearidonic acid, SDA)	0.01 ± 0.02
C11:0 (Undecanoate)	0.00 ± 0.00	C20:2 (Eicosadienoic acid)	0.27 ± 0.06
C12:0 (Laurate)	6.51 ± 2.75	C20:3 n-6 (Dihomo-γ-linolenic acid, DGLA)	0.46 ± 0.13
C13:0 (Tridecanoate)	0.01 ± 0.01	C20:3 n-3 (11,14,17-Eicosatrienoic acid)	0.01 ± 0.01
C14:0 (Myristate)	6.89 ± 3.25	C20:4 n-6 (Arachidonic acid, ARA)	0.46 ± 0.10
C15:0 (Pentadecanoate)	0.20 ± 0.08	C22:2 n-6 (Docosadienoic acid)	0.07 ± 0.02
C16:0 (Palmitate)	25.50 ± 2.49	C20:5 n-3 (Eicosapentaenoic acid, EPA)	0.02 ± 0.01
C17:0 (Heptadecanoate)	0.23 ± 0.08	C22:5 n-3 (Docosapentaenoic acid, DPA)	0.07 ± 0.02
C18:0 (Stearate)	5.29 ± 0.73	C22:6 n-3 (Docosahexaenoic acid, DHA)	0.08 ± 0.04
C20:0 (Arachidate)	0.14 ± 0.04	Total TFA	0.31 ± 0.21
C21:0 (Heneicosanoate)	0.12 ± 0.07	C14:1 (Myristoleic acid, trans)	0.00 ± 0.01
C22:0 (Behenate)	0.06 ± 0.03	C15:1 (Pentadecenoic acid, trans)	0.01 ± 0.02
C23:0 (Tricosanoate)	0.01 ± 0.01	C16:1 (Palmitoleate, trans)	0.07 ± 0.05
C24:0 (Lignocerate)	0.03 ± 0.02	C17:1 (Heptadecenoic acid)	0.00 ± 0.01
Total MUFAs	36.86 ± 4.82	C18:1 n-9 (Elaidic acid)	0.18 ± 0.15
C14:1 (Myristoleic acid)	0.13 ± 0.07	C18:1 (Vaccenate)	0.02 ± 0.04
C16:1 (Palmitoleate)	2.37 ± 1.07	C18:2 n-6 (Linolelaidic acid)	0.01 ± 0.01
C17:1 (Heptadecenoic acid)	0.12 ± 0.09	C19:1 (7-Nonadecenoate, trans)	0.08 ± 0.07
C18:1 n-9 (Oleic acid)	32.30 ± 4.03	C19:1 (10-Nonadecenoate, trans)	0.02 ± 0.04
C18:1 n-7 (Vaccenic acid)	0.77 ± 0.33	C20:1 (Gondoic acid, trans)	0.00 ± 0.00
C20:1 n-9 (Gondoic)	0.29 ± 0.07	Omega-3	0.51 ± 0.19
C22:1 n-9 (Erucate)	0.04 ± 0.02	Omega-6	15.72 ± 3.05
C24:1 n-9 (Nervonate)	0.03 ± 0.01	Omega-9	33.71 ± 4.48
Total PUFA	16.48 ± 3.09	Omega-6/Omega-3	35.97 ± 17.47

Data are presented as Mean ± SD. SFA, saturated fatty acids; MUFA, monounsaturated fatty acids; PUFA, polyunsaturated fatty acids; TFA, trans fatty acids.

### 3.3 Associations between maternal intake and HM composition

#### 3.3.1 24-h dietary recall

Average maternal dietary intake estimated from 24-h dietary recall (Supplementary Table S1). Table 4 and Supplementary Table S2 presents Pearson’s correlation coefficient (r) between maternal nutrient intake estimated from 24-h dietary recall and HM composition. Milk protein concentration showed a positive correlation with maternal MUFA intake (true protein: *r* = 0.384, *p* < 0.05) (Supplementary Table S2). Maternal energy intake (kcal/day) was positively correlated with the percentage of eicosapentaenoic acid (EPA) (C20:5 n-3) in HM (*r* = 0.527, *p* < 0.01) and with total n-3 (% of total FAs) (*r* = 0.489, *p* < 0.01). In contrast, maternal energy intake was inversely correlated with the n-6/n-3 ratio in milk (*r* = −0.410, *p* < 0.01). Maternal trans-fat intake was positively correlated with the percentage of LA (C18:2 n-6) in milk (*r* = 0.417, *p* < 0.01).

**Table 4:** Pearson correlation coefficient (*r*) between maternal dietary intake from 24-h recall and human milk fatty acids (*n* = 40).

Milk content	Maternal dietary intake										
	Energy (kcal/day)	Cho (g/day)	Protein (g/day)	Fat (g/day)	SFA (g/day)	MUFA (g/day)	PUFA (g/day)	Omega-3 (g/day)	Omega-6 (g/day)	Trans fat (g/day)	Omega-6/ Omega-3
C18:2 n-6 LA	0.053	0.075	0.021	−0.039	−0.019	−0.129	0.049	0.245	0.068	**0.417** ^**^	−0.172
C18:3 n-6 (γ-Linolenic acid, GLA)	0.287	0.051	**0.422** ^**^	0.141	0.166	0.108	−0.055	0.056	−0.055	−0.025	−0.010
C18:3 n-3, ALA	**0.359** ^*^	0.306	**0.335** ^*^	0.127	0.004	−0.138	−0.011	0.046	0.019	0.256	0.020
C20:5 n-3, EPA	**0.527** ^**^	0.298	**0.421** ^**^	0.296	**0.354** ^*^	0.182	0.207	0.162	0.219	0.282	0.088
C22:5 n-3, DPA	**0.439** ^**^	0.190	**0.361** ^*^	**0.332** ^*^	**0.368** ^*^	0.227	0.129	0.111	0.152	0.119	0.082
C22:6 n-3, DHA	0.252	0.020	0.189	0.289	0.222	0.299	0.212	0.071	0.251	0.054	0.276
SFAs	−0.205	−0.167	−0.123	−0.090	−0.030	0.059	−0.143	−0.229	−0.123	**−0.322** ^*^	0.069
MUFAs	0.189	0.199	0.124	0.042	0.013	−0.100	0.091	0.082	0.058	0.223	0.022
PUFA	0.033	0.001	0.002	0.027	−0.021	−0.039	0.071	0.286	0.077	0.291	−0.205
TFAs	0.332^*^	0.089	0.192	**0.339** ^*^	0.264	**0.373** ^*^	0.260	0.136	0.288	0.067	0.091
Total Omega-3	**0.489** ^**^	**0.353** ^*^	**0.338** ^*^	0.289	0.167	−0.037	0.109	0.128	0.144	0.225	0.047
Total Omega-6	0.002	−0.020	−0.018	0.005	−0.036	−0.044	0.063	0.279	0.066	0.280	−0.209
Omega-6/ Omega-3	**−0.410** ^**^	−0.330^*^	−0.250	−0.238	−0.173	0.007	−0.093	−0.004	−0.120	−0.093	−0.111

Values are Pearson correlation coefficient (*r*), two tailed tests. Significance: *p* < 0.05 (*) and *p* < 0.01 (**). Bold values indicate statistically significant correlations. Total fat is expressed as g/100 mL; individual fatty acids as % of total fat. SFA, saturated fatty acids; MUFA, monounsaturated fatty acids; PUFA, polyunsaturated fatty acids; LA, Linoleic acid; ALA, α-linolenic acid; EPA, eicosapentaenoic acid; DPA, docosapentaenoic acid; DHA, docosahexaenoic acid.

#### 3.3.2 Omega-3 intake FFQ

**Figure 1:**
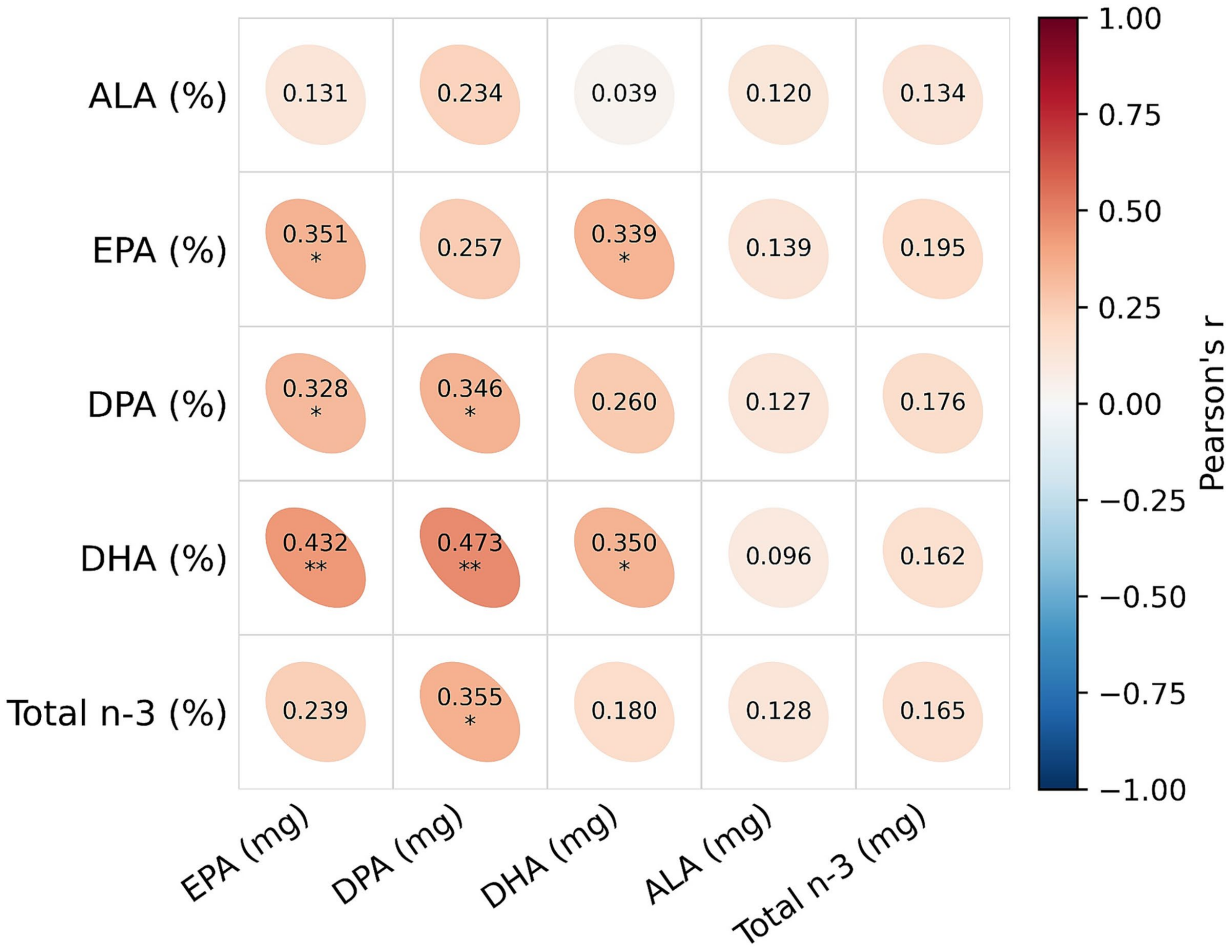
Pearson’s correlation coefficient (*r*) between maternal omega-3 intake estimated using FFQ and omega-3 fatty acids in human milk, *p* < 0.05 (*) and *p* < 0.01 (**). N-3: Omega-3; ALA, *α*-linolenic acid; EPA, eicosapentaenoic acid; DPA, docosapentaenoic acid; DHA, docosahexaenoic acid; FFQ, food frequency questionnaire.

Maternal n-3 intake estimated by FFQ showed positive correlations with n-3 FAs in HM (Figure 1). FFQ DHA intake was positively correlated with milk DHA (r = 0.350, *p* < 0.05) and milk EPA (*r* = 0.339, *p* < 0.05). FFQ docosapentaenoic acid (DPA) intake was positively correlated with milk total n-3 (*r* = 0.355, *p* < 0.05), milk DHA (*r* = 0.473, *p* < 0.01), and milk DPA (*r* = 0.346, *p* < 0.05). In addition, FFQ EPA intake was positively correlated with milk EPA (*r* = 0.351, *p* < 0.05), milk DHA (*r* = 0.432, *p* < 0.01), and milk DPA (*r* = 0.328, *p* < 0.05) (Supplementary Table S3).

#### 3.3.3 Fat intake FFQ

The intake frequency of fat-related food groups is shown in Table 5. Overall, participants reported low intake of fish and seafood, walnuts, flaxseeds, and chia seeds. Table 6 presents Kendall’s rank correlation coefficients between concentration of fat and FAs HM and intake frequency of food products. Maternal walnut intake frequency was positively correlated with milk *α*-linolenic acid (ALA) (τb = 0.343, *p* < 0.01). In addition, maternal egg intake frequency was positively correlated with milk DHA (τb = 0.322, *p* < 0.01), and red meat frequency was positively correlated with total n-3 in milk (τb = 0.342, *p* < 0.01). The n-6/n-3 was positively correlated with red meat intake frequency and negatively correlated with chicken intake frequency (τb = 0.286, *p* < 0.05), (τb = −0.251, *p* < 0.05) respectively.

**Table 5:** Frequency of intake of selected fat- and omega-3–related foods during the previous 6 months (*n* = 40).

Food item	Never	<Once/month	1–3 times/month	1–2 times/week	3–6 times/week	Daily
Fish & seafood	35.00%	30.00%	25.00%	10.00%	0.00%	0.00%
Walnuts	40.00%	17.50%	22.50%	10.00%	2.50%	7.50%
Eggs	7.50%	2.50%	15.00%	22.50%	20.00%	32.50%
Red meat	2.50%	7.50%	37.50%	25.00%	22.50%	5.00%
Chicken	2.50%	0.00%	5.00%	7.50%	32.50%	52.50%
Flaxseeds	90.00%	7.50%	0.00%	0.00%	0.00%	2.50%
Chia seeds	85.00%	7.50%	2.50%	2.50%	0.00%	2.50%
Sunflower oil	47.50%	2.50%	12.50%	5.00%	10.00%	22.50%
Corn oil	57.50%	5.00%	5.00%	7.50%	10.00%	15.00%
Olive oil	12.50%	2.50%	15.00%	15.00%	15.00%	40.00%
Ghee (animal)	22.50%	5.00%	22.50%	17.50%	17.50%	15.00%
Butter	42.50%	15.00%	27.50%	10.00%	5.00%	0.00%

Values are % of participants in each frequency category; rows sum to 100%.

**Table 6:** Kendall’s τb correlations between frequency of fat-related food intake (FFQ) and human milk fat and fatty acid composition (*n* = 40).

Maternal intake	FAT	SFA	MUFA	PUFA	n-3	n-6	DHA	EPA	DPA	ALA	LA	n-6/n-3
Fish & seafood	0.025	−0.110	0.039	0.050	0.221	0.018	0.224	0.182	0.084	0.090	−0.042	0.150
Walnuts	−0.162	−0.092	0.059	−0.053	0.230	−0.050	0.232	0.185	0.280*	0.343**	0.082	0.159
Eggs	0.140	−0.197	0.244*	−0.046	0.237*	−0.062	0.322**	0.286*	0.248*	0.207	0.049	0.204
Red meat	0.013	−0.108	0.176	−0.013	0.342**	−0.040	0.234	0.060	0.101	0.133	0.029	0.286*
Chicken	−0.055	0.109	−0.208	0.051	−0.220	0.078	−0.155	−0.089	0.055	−0.026	0.013	−0.251*
Flaxseeds	−0.109	−0.172	0.267*	−0.095	0.053	−0.107	0.077	0.183	0.074	0.305*	0.050	0.053
Chia seeds	0.156	0.125	−0.113	−0.012	0.086	−0.020	0.213	0.146	0.095	0.083	−0.117	0.049
Sunflower oil	0.093	0.090	−0.085	−0.050	−0.017	−0.055	−0.003	−0.037	−0.146	−0.122	−0.055	−0.024
Corn oil	0.006	−0.191	0.186	0.079	−0.050	0.084	−0.071	0.117	0.132	0.192	0.190	−0.064
Olive oil	−0.035	−0.018	−0.069	0.146	0.026	0.133	0.133	−0.010	0.066	0.031	0.068	−0.039
Ghee (animal)	0.170	0.080	−0.040	−0.140	0.030	−0.155	0.070	0.081	0.141	−0.076	−0.233*	0.102
Butter	0.033	−0.184	0.068	0.157	0.097	0.153	−0.020	0.189	0.180	0.235	0.197	0.014

Values are Kendall’s τb. Significance: *p < 0.05, **p < 0.01. Bold values indicate statistically significant correlations. Milk fat is expressed as g/100 mL; fatty acids are expressed as % of total fatty acids. LA, linoleic acid; ALA, α-linolenic acid; EPA, eicosapentaenoic acid; DPA, docosapentaenoic acid; DHA, docosahexaenoic acid; n-3, omega-3; n-6, omega-6.

### 3.4 Other maternal factors

**Figure 2:**
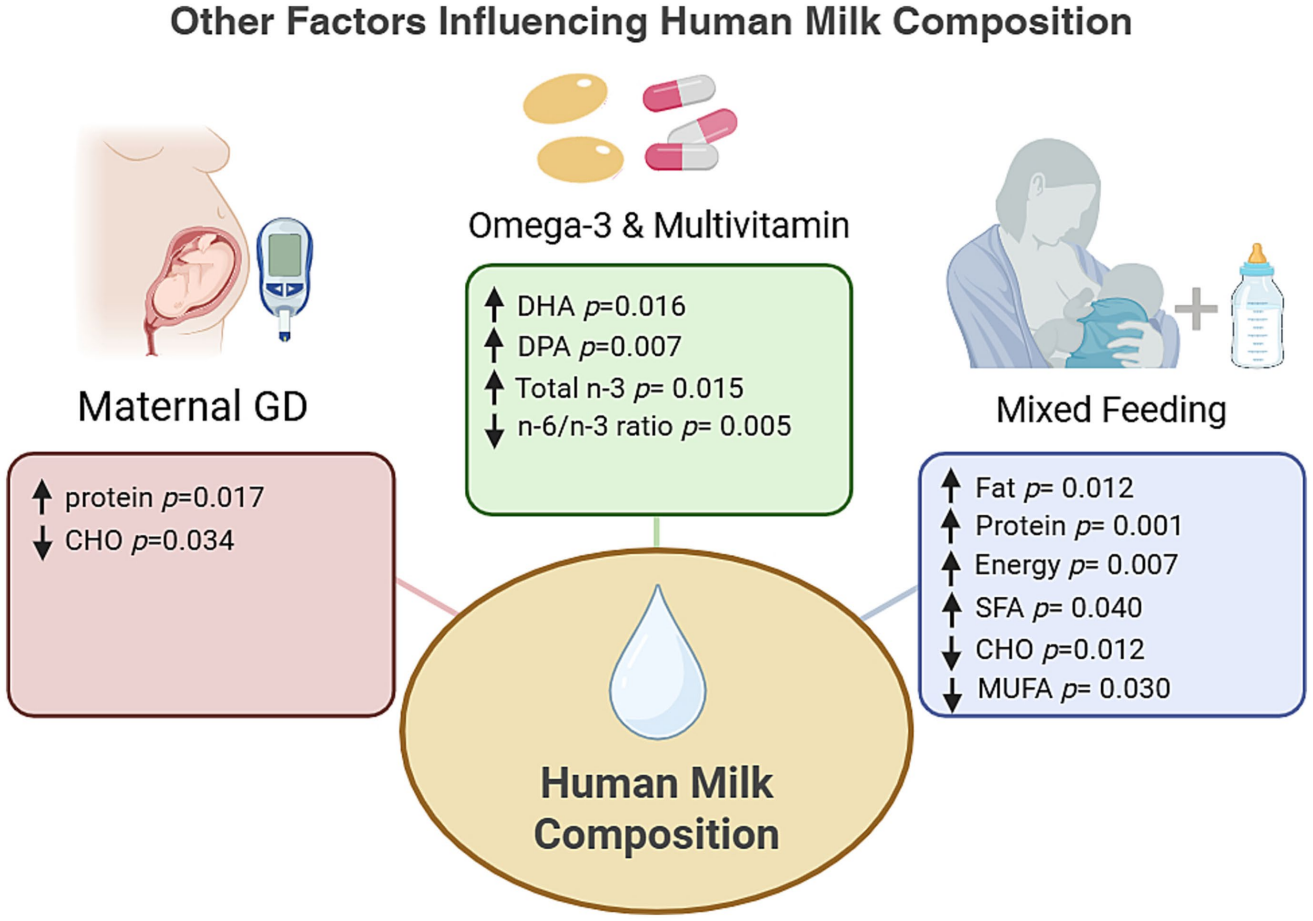
Other factors influencing human milk composition (milk fat, protein, lactose, energy, and selected fatty acids). CHO, carbohydrates; GD, gestational diabetes; DHA, docosahexaenoic acid; DPA, docosapentaenoic acid; SFA, saturated fatty acids; MUFA, monounsaturated fatty acids. *p* values for continuous variables are from Mann–Whitney *U* test. Created by the authors using BioRender (www.biorender.com). A publication license has been obtained. Citation: Created in BioRender. Alodhaybi, S. (2026) https://BioRender.com/wg5yhnk

HM from non-exclusively breastfeeding mothers had higher fat and energy content and a greater proportion of SFA, but lower MUFA proportions and carbohydrate concentrations than milk from exclusively breastfeeding mothers (Figure 2; Supplementary Table S4; all *p* < 0.05). Mothers with GD had higher milk protein and lower carbohydrate concentrations than those without this condition (Supplementary Table S5; *p* < 0.05). Maternal n-3 supplementation was associated with higher total n-3 and a lower n-6/n-3 ratio, and multivitamin use was associated with higher DHA and DPA concentrations (Figure 2; Supplementary Tables S4–S6; *p* < 0.05). No significant differences were observed according to maternal BMI (pre-pregnancy or current) or infant sex.

## 4 Discussion

This study demonstrates that while the macronutrient content of HM among Saudi women remains relatively stable, the FA profile is notably sensitive to maternal dietary intake. In this cohort, we observed that SFAs predominated, followed by MUFAs and PUFAs, while oleic, palmitic, and LA were the most abundant individual components in the HM. A key finding was the strong association between maternal diet and milk composition; maternal intake of n-3 components correlated significantly with their corresponding levels in HM. Additionally, we observed that consuming particular fat-rich foods (e.g., eggs, walnuts, red meat, chicken, and flaxseeds) was linked to distinct FA profiles, including variation in ALA, DPA, and the n-6/n-3 ratio. Beyond FAs, we also found that maternal intake of MUFAs was positively correlated with milk protein concentrations.

In addition to dietary intake, we analyzed multiple maternal and infant factors to assess their effect on HM composition, including maternal BMI (current and pre-pregnancy), maternal supplement intake (n-3 and multivitamin), infant gender, feeding pattern (breastfeeding exclusive vs. non-exclusive), and maternal GD status. Our secondary analyses revealed that breastfeeding exclusivity and GD primarily modulated macronutrient composition, whereas supplement intake significantly influenced FA profile. Additionally, our previous study linked hypothyroidism to altered HM FA composition (17).

Our observed macronutrient concentrations in mature HM strongly align with recent findings from international cohorts (20–22). Notably, our data align closely with the global reference values recently established by the Mothers, Infants, and Lactation Quality (MILQ) study (23). However, the total fat concentration in our study (3.93 g/100 mL) was slightly higher than the MILQ reference range of (3.4–3.8 g/100 mL). Diurnal variations and the inclusion of non-exclusively breastfeeding mothers likely drove this elevation, because both factors influence lipid density in HM (6, 24).

Consistent with global FA profiles, palmitic acid (C16:0) and oleic acid (C18:1n-9) were the predominant FAs in our cohort, collectively accounting for the majority of total milk fat. While the overall distribution of SFAs and MUFAs followed global trends, total PUFA levels—including both n-6 and n-3 series—were lower than the corresponding international averages. Similarly, TFAs content was notably lower than the global mean (25, 26). Furthermore, our SFA values fell below those reported in Saudi women nearly three decades ago (56.5%) (27), suggesting a nutritional transition. This difference may reflect broader societal and dietary changes in Saudi Arabia over time, including shifts in food availability, increased consumption of processed and convenience foods, changes in culinary practices, and evolving dietary patterns associated with urbanization (28, 29). It is worth noting that while some populations exhibit higher MUFA proportions, specific regional dietary habits explain this variation, such as the Mediterranean diet (8, 13).

Our results indicate a strong positive association between maternal DHA intake and DHA levels in HM. This finding aligns with previous evidence showing that milk DHA composition reflects maternal dietary intake, as well as DHA status in adipose tissue and plasma (9, 11, 12, 16, 30–32). Moreover, habitual dietary patterns rich in n-3 FAs—particularly when established before pregnancy and maintained through lactation—appear to be associated with stronger correlations between intake and milk DHA levels (9, 11, 33).

The low concentrations of n-3 FAs, including DHA, observed in our study likely reflect the cohort’s dietary pattern, characterized by infrequent consumption of fish and seafood (Table 5). Specifically, only 10% of the participants (*n* = 4 out of 40) reported consuming fish and seafood 1–2 times weekly, while (*n* = 14) reported no fish and seafood consumption during the preceding 6 months. Notably, both n-3 intake (including DHA) and HM n-3 levels in our cohort were below global average values, further suggesting suboptimal dietary n-3 exposure (25). This pattern is consistent with reports from Saudi (including Riyadh/inland) populations indicating relatively low seafood intake compared with recommended dietary targets (34, 35). These findings highlight a nutritional gap in marine-derived FAs, which directly influence the FA profile of HM in this region.

Analysis of the habitual intake using the fat-rich foods FFQ suggests that these foods significantly shape the milk FA profile of Saudi mothers. In particular, more frequent walnut consumption was positively associated with ALA levels in milk, while more frequent egg consumption was positively associated with DHA levels in milk. These findings partially contrast with those reported by Aumeistere et al. (31) and Bzikowska-Jura et al. (11), who did not observe similarly specific associations. However, those studies often combined nuts and seeds into a single food group, which may have obscured the distinct contribution of walnuts as a dietary source of ALA (18).

Animal-protein sources also appeared to differentially influence the balance of FAs in the milk. Red meat intake frequency was positively correlated with total n-3 in HM, but it was also associated with a higher n-6/n-3 ratio, suggesting a less favorable overall FA balance. Because we did not identify the specific ruminant species consumed, this may have contributed to the observed variability, since different ruminant meats can differ in FA composition (36). This is particularly relevant in Saudi Arabia, where camel and lamb are commonly preferred over beef (37). In contrast, chicken intake frequency was negatively correlated with the n-6/n-3 ratio, indicating that animal-protein sources may relate differently to maternal FA status and, in turn, to milk lipid composition.

In addition, only 6 of 40 mothers reported n-3 supplementation during pregnancy. Nevertheless, supplementation was associated with higher milk n-3 content and a lower n-6/n-3 ratio, suggesting an overall shift toward a more favorable FA profile. This pattern is partially consistent with prior studies; however, in our sample, DHA, EPA, and DPA increased with supplementation but did not reach statistical significance, potentially attributed to the small number of supplement users, dose variability, or limited statistical power. Notably, DHA has been reported to exhibit a dose–response relationship with milk DHA content, supporting the plausibility that higher or more sustained intake may be required to produce measurable increases in HM DHA (14, 38, 39).

Several observational studies suggest that maternal diet does not strongly influence HM macronutrient particularly protein and lactose. However, meta-analyses and intervention studies indicate that increasing maternal fat intake can increase HM fat, supporting the view that milk fat is the most diet-responsive component. Consequently, diet–macronutrient associations are often inconsistent across studies, which may explain why we did not observe patterns comparable to the literature (8, 10, 29, 40–44, 7–). In our analysis, maternal MUFA intake was the only dietary factor associated with a total (true) milk protein. Studies rarely report MUFA–protein findings, thus, the result should be interpreted cautiously. We also found a positive correlation between energy, carbohydrate, protein, and fat intake and several n-3 FAs. This was supported by Munhoz et al. (45), who reported that total dietary intake correlated with total n–3 PUFAs and their components, suggesting that higher overall intake may reflect greater dietary variety and, in turn, higher n-3 FA availability for incorporation into HM. Additionally, we found a positive correlation between TFA intake and LA; this result contradicts previous findings showing a reduction in essential FAs in HM with higher maternal TFA intake (46, 47).

Milk from non-exclusively breastfeeding mothers contained higher levels of fat, energy, protein, and SFAs, and lower levels of MUFAs and carbohydrates. These findings are partially consistent with previous studies reporting higher protein and lower carbohydrate concentrations in milk from non-exclusive breastfeeders (20, 48). Ni et al. also observed higher fat and energy levels at 60 days postpartum, although these differences did not reach statistical significance in their study. Mothers with GD in our cohort exhibited higher protein and lower carbohydrate concentrations in their milk. A recent systematic review and meta-analysis partially support these findings, reporting higher protein levels in the breast milk of mothers with GD (49). However, evidence on the impact of maternal GD on HM composition remains conflicting (50–52), indicating that additional well-designed studies are needed. Notably, all six mothers with GD in this cohort were managed through dietary modification alone, which may have minimized pharmacological confounding on milk composition. Nevertheless, given the small number of GD cases (*n* = 6), these findings should be interpreted with considerable caution and are considered exploratory.

The inclusion of mothers with controlled hypothyroidism and GD, together with the high prevalence of overweight and obesity, should be considered when interpreting these findings, as these factors may influence baseline HM composition. Notably, although maternal obesity has been linked to altered FA profiles in previous studies, this was not observed in the present cohort (53, 54).

Our results also indicate that maternal multivitamin supplementation significantly improved the n-3 status of HM, as reflected by higher concentrations of DHA and DPA. This effect may be related to the overall better dietary quality typically observed in supplement users (55). It is noteworthy that all participants in our study were prescribed multivitamins for 60 days before discharge.

This study offers updated and comprehensive data on HM composition among Saudi women, integrating both macronutrient measures and an extensive FA profile. Maternal dietary intake was captured using multiple complementary tools, including repeated 24-h dietary recalls, a fat-rich FFQ, and an n-3–specific FFQ, strengthening dietary exposure assessment. A key methodological strength was the collection of the complete contents of one breast, which captures both foremilk and hindmilk and therefore provides a more representative estimate of the total nutrient density available to the infant. In addition, evaluating multiple maternal characteristics (e.g., anthropometry, health status, breastfeeding pattern, and supplement use) improved our ability to identify determinants of HM variability. To our knowledge, this study is the first study in Saudi Arabia to characterize FA and macronutrient compositions of HM. Furthermore, this is the first to comprehensively examine associations between both current and habitual maternal dietary intake on HM composition in this region.

However, several limitations should be considered when interpreting these findings. This study represents a secondary analysis of a larger cohort, and no *a priori* power calculation was performed. Given the large number of associations tested across multiple FAs and maternal exposures without adjustment for multiple comparisons, the risk of Type I error cannot be excluded; therefore, the findings should be interpreted as exploratory and confirmed in larger, purpose-designed studies. The relatively small and heterogeneous sub-sample — including mothers with controlled hypothyroidism (*n* = 9), gestational diabetes (*n* = 6), and a high prevalence of overweight and obesity (72.5%) — may introduce variability in lipid metabolism and baseline FA profiles, as well as residual confounding that could not be fully accounted for. HM composition was assessed at a single time point; therefore, longitudinal changes, infant outcomes, and validation using maternal serum FA biomarkers could not be evaluated. Cultural and logistical constraints limited sampling to a single collection rather than 24-h sampling, which may have obscured diurnal variation in HM composition. Additionally, dietary intake assessment relied on 24-h recalls, which are subject to recall bias and may not fully represent habitual intake despite repeated measurements. Furthermore, dietary analysis was conducted using ESHA Food Processor software based on the USDA National Nutrient Database, which may not fully capture traditional Saudi foods, as a validated national food composition database is currently unavailable in Saudi Arabia.

## 5 Conclusion

The most consistent finding across our data and the existing literature is a positive association between maternal n-3 intake and n-3 content in HM. In our cohort, approximately 15% of mothers demonstrated zero intake of n-3 FAs based on FFQ-derived dietary analysis, while a substantial proportion reported no consumption of foods rich in n-3 FAs during the preceding 6 months; this may be reflected in the low n-3 content and elevated n-6/n-3 ratio observed in their milk. These observations suggest the potential role of educating pregnant and lactating women about n-3 FAs and appropriate dietary sources. Previous studies have shown that providing targeted information to mothers can increase the n-3 content of their milk (56). In Saudi Arabia, many women reduce or avoid fish during pregnancy—sometimes based on advice from healthcare providers or family members regarding the potential risks of mercury and heavy metal contamination in certain fish species. This concern may lead to an unnecessarily strong reduction in marine n-3 sources, particularly in inland populations such as our cohort (57, 58). Therefore, counseling may need to address safe fish choices during pregnancy while also emphasizing non-marine n-3 sources such as walnuts, flaxseed, and chia seeds. Given that our cohort represents the first comprehensive assessment of HM macronutrients in Saudi Arabia, further exploratory and longitudinal research is warranted to better understand the unique biological and environmental determinants of milk composition in this understudied population.

## Data Availability

The datasets used in this study are not publicly available due to privacy and ethical restrictions. Requests to access the data should be directed to the corresponding authors, Shahad Alodhaybi at Shahadalodhaybi@gmail.com or Azza Madkhali at madkhalia@mngha.med.sa.
