# Supplementary material for: Association of maternal diet with human milk fatty acid and macronutrient composition: a Saudi cohort study
Source: Front Nutr. 2026 May 4;13:1830854. doi: 10.3389/fnut.2026.1830854 (PMC13180842; doi:10.3389/fnut.2026.1830854)
Supplement: Supplementary file 1 [file Table_1.docx]

**Supplementary materials**

**Table S1: Average maternal dietary intake from 24 h recall between mothers with and without hypothyroidism.** Data are mean ± SD. SFA, saturated fatty acids; MUFA, monounsaturated fatty acids; PUFA, polyunsaturated fatty acids.

| **Dietary intake** | **Mean ±** **SD** |
| --- | --- |
| Energy (kcal/day) | 1693.8 ± 333.029 |
| Carbohydrates (g/day) | 199.4 ± 52.1 |
| Protein (g/day) | 68.6 ± 35.8 |
| Fat (g/day) | 72.17 ±18.927 |
| SFA (g/day) | 21.2 ±7.7 |
| MUFA (g/day) | 20.2 ± 6.6 |
| PUFA (g/day) | 14.1 ± 6.0 |
| Omega-3 (g/day) | 1.1± 0.5 |
| Omega-6 (g/day) | 12.1 ± 5.6 |
| Omega-6/ omega-3 | 12.1 ± 5.4 |
| Trans fat (g/day) | 0.251 ± 0.260 |
| Cholesterol (mg/ day) | 272.9 ± 123.6 |
| Fat (kcal/day) | 653.1 ± 164.9 |
| SFA (kcal/day) | 191.0 ± 69.7 |

| Human milk macronutrient composition | Maternal dietary intake | | | | | | | | | | |
| --- | --- | --- | --- | --- | --- | --- | --- | --- | --- | --- | --- |
|  | Energy (kcal/day) | Cho (g/day) | Protein (g/day) | Fat (g/day) | SFA (g/day) | MUFA (g/day) | PUFA (g/day) | Omega-3 (g/day) | Omega-6 (g/day) | Trans fat (g/day) | Omega-6/ omega-3 |
| FAT (g/100ml) | 0.110 | 0.276 | -0.098 | 0.042 | 0.012 | -0.068 | -0.046 | -0.149 | -0.042 | 0.094 | 0.091 |
| Crude Protein (g/100ml) | 0.090 | -0.051 | -0.018 | 0.281 | 0.227 | **.383^*^** | 0.045 | 0.007 | 0.101 | -0.096 | 0.016 |
| CHO (g/100ml) | -0.041 | -0.115 | 0.053 | -0.045 | 0.104 | 0.001 | 0.024 | 0.103 | -0.025 | 0.297 | -0.056 |
| Total solids (g/100ml) | 0.067 | 0.205 | -0.113 | 0.044 | 0.040 | -0.053 | -0.054 | -0.129 | -0.051 | 0.107 | 0.069 |
| Energy | 0.103 | 0.247 | -0.100 | 0.060 | 0.042 | -0.035 | -0.044 | -0.138 | -0.039 | 0.106 | 0.083 |
| True Protein (g/100ml) | 0.092 | -0.048 | -0.017 | 0.281 | 0.228 | **.384^*^** | 0.045 | 0.006 | 0.100 | -0.096 | 0.017 |
| Values are mean ± SD. Bold values indicate statistically significant correlations (p < 0.05). Cho, carbohydrate; SFA, saturated fatty acids; MUFA, monounsaturated fatty acids; PUFA, polyunsaturated fatty acids. | | | | | | | | | | | |

**Table S2: Pearson correlation coefficient (r) between maternal dietary intake from 24-h recall and human milk macronutrients (n=40).**

**Table S3: Pearson’s correlation coefficient (r) between maternal daily omega-3 intake estimated by FFQ and omega-3 fatty acids in human milk (n=40).** P<0.05 (*) and P<0.01 (**). N-3: omega-3; ALA, α-linolenic acid; EPA, eicosapentaenoic acid; DPA, docosapentaenoic acid; DHA, docosahexaenoic acid; FFQ, food frequency questionnaire.

| **Omega-3 fatty acids in human milk (%)** | **Maternal dietary intake** | | | | | |
| --- | --- | --- | --- | --- | --- | --- |
|  | **FFQ EPA mg** | **FFQ DPA mg** | **FFQ DHA mg** | **FFQ ALA mg** | **FFQ N-3 TOTAL mg** | |
| **DHA** | **.432^**^** | **.473^**^** | **.350^*^** | 0.096 | | 0.162 |
| **DPA** | **.328^*^** | **.346^*^** | 0.260 | 0.127 | | 0.176 |
| **EPA** | **.351^*^** | 0.257 | **.339^*^** | 0.139 | | 0.195 |
| **ALA** | 0.131 | 0.234 | 0.039 | 0.120 | | 0.134 |
| **total Omega-3** | 0.239 | **.355^*^** | 0.180 | 0.128 | | 0.165 |

| **Human milk components** | **With GD (n=6)** | **Without GD (n=34)** | p-value |
| --- | --- | --- | --- |
| **Macronutrients (per 100 mL)** | | | |
| **FAT (g/100ml)** | 4.042 ± 1.499 | 3.909 ± 1.683 | 0.315 |
| **Carbohydrate (g/100ml)** | 7.355 ± 0.550 | 7.762 ± 0.489 | **0.034** |
| **True Protein (g/100ml)** | 1.270 ± 0.324 | 1.029 ± 0.419 | **0.017** |
| **Energy (kcal/100ml)** | 73.803 ± 13.354 | 72.717 ± 15.895 | 0.384 |
| **Fatty acids (%)** | | | |
| **EPA (%)** | 0.013 ± 0.014 | 0.017 ± 0.010 | 0.373 |
| **DPA (%)** | 0.056 ± 0.028 | 0.068 ± 0.024 | 0.218 |
| **DHA (%)** | 0.082 ± 0.046 | 0.082 ± 0.040 | 0.880 |
| **SFAs (%)** | 48.640 ± 8.670 | 45.246 ± 5.629 | 0.289 |
| **MUFAs (%)** | 34.633 ± 7.390 | 37.256 ± 4.259 | 0.495 |
| **PUFAs (%)** | 15.929 ± 3.017 | 16.582 ± 3.131 | 0.705 |
| **TFAs (%)** | 0.309 ± 0.329 | 0.314 ± 0.189 | 0.544 |
| **total Omega-3 (%)** | 0.467 ± 0.184 | 0.517 ± 0.190 | 0.449 |
| **total Omega-6 (%)** | 15.190 ± 3.011 | 15.811 ± 3.089 | 0.705 |
| **Omega-6/ Omega-3 ratio** | 37.616 ± 17.279 | 35.675 ± 17.744 | 0.596 |
| Values are presented as Mean ± SD. Group comparisons are reported as p-values (two-sided) from Mann–Whitney U test. Statistically significant p-values (<0.05) are shown in bold. Abbreviations: SFA, saturated fatty acids; MUFA, monounsaturated fatty acids; PUFA, polyunsaturated fatty acids; TFA, trans fatty acids; EPA, eicosapentaenoic acid; DPA, docosapentaenoic acid; DHA, docosahexaenoic acid. | | | |

**Table S4. Human milk composition by gestational diabetes status.**

**Table S5. Human milk composition by feeding pattern.**

| **Human milk components** | **Exclusive breastfeeding (n=16)** | **Mixed feeding (n=24)** |  |
| --- | --- | --- | --- |
| **Macronutrients (per 100 mL)** | | | |
| **FAT (g/100ml)** | 3.215 ± 1.565 | 4.404 ± 1.539 | **0.012** |
| **Carbohydrate (g/100ml)** | 7.846 ± 0.513 | 7.605 ± 0.500 | **0.012** |
| **True Protein (g/100ml)** | 0.878 ± 0.166 | 1.190 ± 0.479 | **0.001** |
| **Energy (kcal/100ml)** | 65.919 ± 14.729 | 77.520 ± 14.278 | **0.007** |
| **Fatty acids (%)** | | | |
| **EPA (%)** | 0.019 ± 0.012 | 0.015 ± 0.008 | 0.194 |
| **DPA (%)** | 0.069 ± 0.028 | 0.064 ± 0.023 | 0.516 |
| **DHA (%)** | 0.078 ± 0.036 | 0.085 ± 0.043 | 0.793 |
| **SFAs (%)** | 43.748 ± 6.908 | 47.093 ± 5.345 | **0.040** |
| **MUFAs (%)** | 38.878 ± 5.355 | 35.520 ± 4.001 | **0.030** |
| **PUFAs (%)** | 16.536 ± 3.549 | 16.449 ± 2.815 | 0.669 |
| **TFAs (%)** | 0.293 ± 0.229 | 0.327 ± 0.200 | 0.400 |
| **total Omega-3 (%)** | 0.559 ± 0.208 | 0.477 ± 0.170 | 0.172 |
| **total Omega-6 (%)** | 15.745 ± 3.407 | 15.700 ± 2.860 | 0.649 |
| **Omega-6/ Omega-3 ratio** | 32.060 ± 15.350 | 38.571 ± 18.605 | 0.327 |
| Values are presented as Mean ± SD. Group comparisons are reported as p-values (two-sided) from Mann–Whitney U test. Statistically significant p-values (<0.05) are shown in bold. Abbreviations: SFA, saturated fatty acids; MUFA, monounsaturated fatty acids; PUFA, polyunsaturated fatty acids; TFA, trans fatty acids; EPA, eicosapentaenoic acid; DPA, docosapentaenoic acid; DHA, docosahexaenoic acid. | | | |

**Table S6. Human milk macronutrient (per 100 mL) and fatty acid profile (%) according to maternal supplement use.**

**Panel A:** Omega-3 supplement use during pregnancy

| **Human milk components** | **Yes omega-3 (n=6)** | **No omega-3 (n=34)** |  |
| --- | --- | --- | --- |
| **Macronutrients (per 100 mL)** | | | |
| **FAT (g/100ml)** | 3.367 ± 1.316 | 4.028 ± 1.687 | 0.472 |
| **Carbohydrate (g/100ml)** | 7.443 ± 0.794 | 7.747 ± 0.448 | 0.460 |
| **True Protein (g/100ml)** | 1.080 ± 0.204 | 1.062 ± 0.441 | 0.264 |
| **Energy (kcal/100ml)** | 66.875 ± 11.613 | 73.940 ± 15.865 | 0.384 |
| **Fatty acids (%)** | | | |
| **EPA %** | 0.018 ± 0.009 | 0.016 ± 0.011 | 0.393 |
| **DPA %** | 0.087 ± 0.036 | 0.063 ± 0.021 | 0.139 |
| **DHA %** | 0.090 ± 0.040 | 0.080 ± 0.040 | 0.622 |
| **SFAs %** | 48.439 ± 7.862 | 45.281 ± 5.827 | 0.353 |
| **MUFAs %** | 35.860 ± 5.557 | 37.040 ± 4.752 | 0.925 |
| **PUFAs %** | 14.828 ± 3.019 | 16.776 ± 3.047 | 0.167 |
| **TFAs %** | 0.244 ± 0.146 | 0.326 ± 0.219 | 0.507 |
| **total Omega-3 %** | 0.685 ± 0.172 | 0.479 ± 0.175 | **0.015** |
| **total Omega-6 %** | 13.906 ± 2.877 | 16.037 ± 3.004 | 0.145 |
| **Omega-6/ Omega-3 %** | 21.112 ± 5.031 | 38.588 ± 17.597 | **0.005** |
| Values are presented as Mean ± SD. Group comparisons are reported as p-values (two-sided) from Mann–Whitney U test. Statistically significant p-values (<0.05) are shown in bold. Abbreviations: SFA, saturated fatty acids; MUFA, monounsaturated fatty acids; PUFA, polyunsaturated fatty acids; TFA, trans fatty acids; EPA, eicosapentaenoic acid; DPA, docosapentaenoic acid; DHA, docosahexaenoic acid.  “Omega-3 use” defined as maternal intake of omega-3 supplements during pregnancy (yes/no, based on questionnaire). | | | |

**Panel B.** Multivitamin use in the preceding last 4 weeks before milk sample

| **Human milk components** | **Yes multivitamin (n=21)** | **No multivitamin (n=19)** |  |
| --- | --- | --- | --- |
| **Macronutrients (per 100 mL)** | | | |
| **FAT (g/100ml)** | 4.335 ± 2.036 | 3.479 ± 0.902 | 0.151 |
| **Carbohydrate (g/100ml)** | 7.767 ± 0.455 | 7.628 ± 0.574 | 0.357 |
| **True Protein (g/100ml)** | 1.139 ± 0.517 | 0.984 ± 0.240 | 0.416 |
| **Energy (kcal/100ml)** | 77.209 ± 19.018 | 68.095 ± 8.072 | 0.072 |
| **Fatty acids (%)** | | | |
| **EPA %** | 0.017 ± 0.010 | 0.016 ± 0.011 | 0.416 |
| **DPA %** | 0.075 ± 0.025 | 0.057 ± 0.022 | **0.007** |
| **DHA %** | 0.097 ± 0.045 | 0.065 ± 0.025 | **0.016** |
| **SFAs %** | 46.152 ± 6.971 | 45.316 ± 5.279 | 0.839 |
| **MUFAs %** | 36.741 ± 5.630 | 36.997 ± 3.888 | 0.797 |
| **PUFAs %** | 16.199 ± 2.923 | 16.798 ± 3.307 | 0.735 |
| **TFAs %** | 0.312 ± 0.197 | 0.315 ± 0.229 | 0.839 |
| **total Omega-3 %** | 0.527 ± 0.172 | 0.491 ± 0.207 | 0.323 |
| **total Omega-6 %** | 15.416 ± 2.915 | 16.051 ± 3.234 | 0.655 |
| **Omega-6 / Omega-3%** | 33.908 ± 17.534 | 38.241 ± 17.586 | 0.316 |
| Values are presented as Mean ± SD. Group comparisons are reported as p-values (two-sided) from Mann–Whitney U test. Statistically significant p-values (<0.05) are shown in bold. Abbreviations: SFA, saturated fatty acids; MUFA, monounsaturated fatty acids; PUFA, polyunsaturated fatty acids; TFA, trans fatty acids; EPA, eicosapentaenoic acid; DPA, docosapentaenoic acid; DHA, docosahexaenoic acid.  “**Multivitamin use**” defined as maternal intake of multivitamin supplements in the last 4 weeks (during lactation) (yes/no). | | | |
